# Promoting long-term inhibition of human fear responses by non-invasive transcutaneous vagus nerve stimulation during extinction training

**DOI:** 10.1038/s41598-020-58412-w

**Published:** 2020-01-30

**Authors:** Christoph Szeska, Jan Richter, Julia Wendt, Mathias Weymar, Alfons O. Hamm

**Affiliations:** 1 University of Greifswald, Department of Physiological and Clinical Psychology/Psychotherapy, Franz-Mehring-Strasse 47, 17487 Greifswald, Germany; 2 University of Potsdam, Department of Biological Psychology and Affective Science, Karl-Liebknecht Strasse 24/25, 14476 Potsdam, Germany

**Keywords:** Electromyography - EMG, Amygdala, Extinction, Fear conditioning, Autonomic nervous system

## Abstract

Inhibiting fear-related thoughts and defensive behaviors when they are no longer appropriate to the situation is a prerequisite for flexible and adaptive responding to changing environments. Such inhibition of defensive systems is mediated by ventromedial prefrontal cortex (vmPFC), limbic basolateral amygdala (BLA), and brain stem locus-coeruleus noradrenergic system (LC-NAs). Non-invasive, transcutaneous vagus nerve stimulation (tVNS) has shown to activate this circuit. Using a multiple-day single-cue fear conditioning and extinction paradigm, we investigated long-term effects of tVNS on inhibition of low-level amygdala modulated fear potentiated startle and cognitive risk assessments. We found that administration of tVNS during extinction training facilitated inhibition of fear potentiated startle responses and cognitive risk assessments, resulting in facilitated formation, consolidation and long-term recall of extinction memory, and prevention of the return of fear. These findings might indicate new ways to increase the efficacy of exposure-based treatments of anxiety disorders.

## Introduction

Learning to inhibit defensive responses to cues which are no longer associated with aversive events is crucial for flexible and appropriate responding towards changing environments^1^. During such extinction learning the previously acquired association is not erased from memory, rather the organism has to learn to actively inhibit the previously acquired response – in this case – a fear response^2,3^. There has been extensive research to delineate the neural systems underlying this inhibitory learning process. These efforts have pointed out the crucial role of two interconnected brain regions inhibiting the activity of the central nucleus of the amygdala, the key structure orchestrating fear responses:^4–6^ the basolateral complex of the amygdala (BLA) and the ventromedial prefrontal cortex (vmPFC)^4–11^. While activity of the BLA is involved in the acquisition and consolidation of extinction memory^5,6,12^, increased activity of the vmPFC is found during recall of extinction memory, pinpointing its importance for the long-term inhibition of defensive responses^13,14^.

Invasive peripheral stimulation of the vagus nerve leads to specific noradrenergic activation of both the BLA and vmPFC, by way of activating its afferent projections via the nucleus tractus solitarius to the locus coeruleus-noradrenergic system (LC-NAs)^15–17^. Consequently, invasive stimulation of vagal afferents resulted in enhanced inhibition of defensive freezing during fear extinction learning and promoted extinction memory consolidation and recall in rodents^18–20^, presumably by increasing the activity in the BLA-vmPFC pathway^18,20^. Transcutaneous vagus nerve stimulation (tVNS) serves as a non-invasive equivalent, activating the same brain regions as its invasive counterpart by stimulation of the cymba conchae of the human auricle – a skin area exclusively innervated by the vagus nerve^21,22^. However, transfer from the promising animal findings to humans was only partly successful. While tVNS enhanced extinction of cognitive risk assessments in humans (US expectancy ratings)^23,24^, no effect of tVNS was found for behavioral or physiological measures of fear^23–25^. One reason for these mixed findings might be, that previous human research used the same fixed and rather low tVNS intensities for each participant^23–25^, not taking individual differences in sensitivity into account that might have resulted in reduced stimulation of the vagus nerve in some subjects. More important, most of these human studies used differential-cue conditioning tasks, while aversive single-cue conditioning was used in animal research. Differential-cue conditioning, however, requires more complex discriminative learning during both acquisition and extinction probably involving other neural circuitry^26–28^.

Thus, in the current study we investigated the impact of individually adjusted tVNS on extinction learning in humans using such a single-cue conditioning and extinction paradigm, closely adapted to previous animal research^18,19^. This protocol requires between-subject comparisons of conditioned responses between a fear learning group, in which the CS is repeatedly paired with the unconditioned stimulus (US) and a control group, in which the CS is presented explicitly unpaired with the US^27,29^. Consequently, participants of the fear learning group are expected to acquire conditioned fear responses to the CS, which should extinguish during extinction training. Participants of the control group are expected not to show any conditioned responses to the CS^30^, thus being unaffected by an extinction training. Consequently, this single-cue conditioning design allows to distinguish conditioned fear, which should only develop in the fear learning group, from elevated general defensive alertness due to the mere presentation of the US, which may also be observed in the control group. Hence, the design further allows to disentangle potential fear reducing effects of tVNS due to facilitated extinction of conditioned fear, which should only be observed in the fear learning group. In contrast a reduction of general defensive alertness by tVNS, should be also observed in the control group.

We presumed tVNS to improve the extinction of cognitive and behavioral indices of fear only in the fear learning group, but not in the control group. Moreover, based on animal findings, we hypothesized that tVNS would also facilitate the recall of fear extinction memory in the fear learning group, but not in the control group^19^. Finally, we also wanted to investigate whether tVNS would also reduce the return of fear after reinstatement. Indeed, even a fully extinguished fear response can be reinstated after re-experiencing the threatening event^2,31,32^, likely contributing to relapses after successful exposure therapy^33,34^. If tVNS would reduce reinstatement of fear, long-term efficacy of exposure-based treatments might be augmented using this neuroscience-based intervention.

## Results

**Figure 1:**
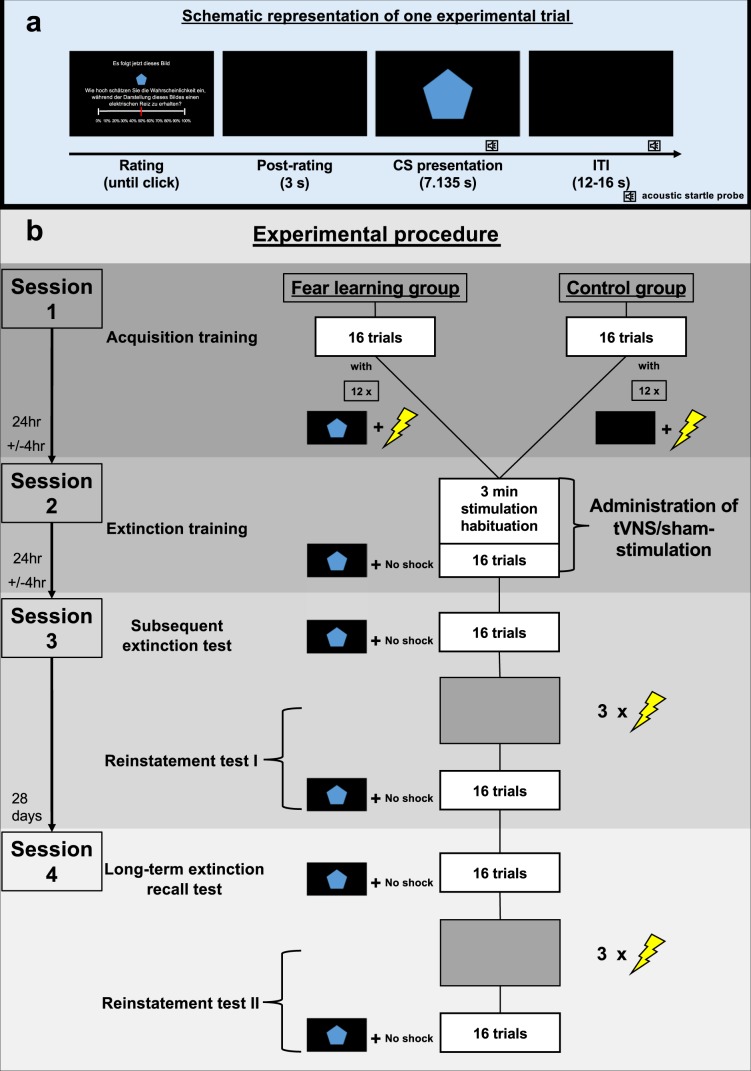
Schematic representation of an experimental trial and the experimental procedure of the single-cue fear conditioning and extinction paradigm. Upper Panel (**a**): Schematic representation of an experimental trial. Each trial started with a shock expectancy rating, where the CS was presented in smaller size and participants were instructed to rate the probability that this cue would be followed by the shock during the upcoming trial (English translation of the German instruction: “Next, this picture will follow. How likely do you think is it, to receive an electrical shock during the upcoming presentation of this picture?”). This procedure is very much comparable to the clinical procedure during exposure therapy, during which the patient is first asked to rate the probability that the central concern might become true (e.g., fainting) before the exposure exercise begins. Three seconds after completing the rating, the cue is presented in full size on the screen, making sure that the physiological fear response is not affected by any simultaneous cognitive evaluation task. Acoustic startle probes were presented both during the CS and ITIs. Lower Panel (**b**): Schematic representation of the experimental procedure. During each experimental phase sixteen trials were presented. During acquisition training participants of the fear learning group received twelve paired presentations of the conditioned stimulus and an electrical shock, while in the control group CS and shocks were presented explicitly unpaired (shocks only during inter-trial intervals). During extinction training, the subsequent extinction test and long-term extinction recall test no electrical shocks were presented. Both reinstatement tests started with three non-signaled electrical shocks, followed by sixteen unreinforced CS presentations. Acoustic startle probes were presented during the CS and ITIs throughout each experimental phase.

TVNS was applied in a 2 × 2 between-group design with fear learning (group: fear learning vs. control) and type of stimulation (stimulation: stimulation of the cymba conchae [tVNS] vs. sham stimulation of the earlobe) as two between subject factors (for a linear depiction of the experimental phases please see Fig. 1).

During acquisition training (**session 1**) subjects of the fear learning group (*n* = 40) received sixteen paired presentations of a single visual cue (conditioned stimulus, CS; geometric figure on a black background) with an aversive electrical shock (unconditioned stimulus, US) during 12 of the 16 trials (75% reinforcement rate). In the control group (*n* = 40) the CS was also presented sixteen times but explicitly unpaired with the US, which was presented 12 times during the inter-trial intervals (ITI; black screen).

Extinction training started 24 hours later (**session 2**), where the CS was now presented without the US in all groups. Half of the subjects of each group underwent extinction training receiving individually adjusted transcutaneous stimulation of the cymba conchae (**tVNS**; *n* = 20 of the fear learning and n = 20 of the control group), while the other half of both groups was given individually adjusted sham stimulation of the earlobe, not stimulating any vagal afferents^22^ (see Fig. 1). Allocation to the stimulation condition (tVNS vs. sham stimulation) was randomized and its administration single-blind sham controlled (see also **Methods**).

Twenty-four hours after extinction training (**session 3**), subjects underwent a subsequent extinction test, during which the CS was presented 16 times without the US, followed by a reinstatement procedure (US was presented alone on three successive trials) and a reinstatement test session (16 CS presentations without US) to investigate the effects of tVNS on short-term extinction memory recall and the effect of tVNS on reinstatement of fear.

Long-term effects of tVNS were tested approximately 28 days after the third assessment (**session 4**), using the same procedure as during session 3.

We assessed shock expectancy ratings as an index of cognitive risk assessments and the potentiation of the startle-eyeblink reflex elicited by a white noise probe stimulus (95 dB, 50 ms) – a low level brainstem reflex reflecting fear in humans and other animals as it is indexing amygdala-dependent automatic freezing^35–38^. Correspondingly, potentiation of startle responses during CS trials compared to ITIs reflects the level of fear elicited by the CS.

### Between-group single-cue fear conditioning results in stable conditioned fear responses in a fear learning group, but not in a control group (session 1; acquisition training)

**Figure 2:**
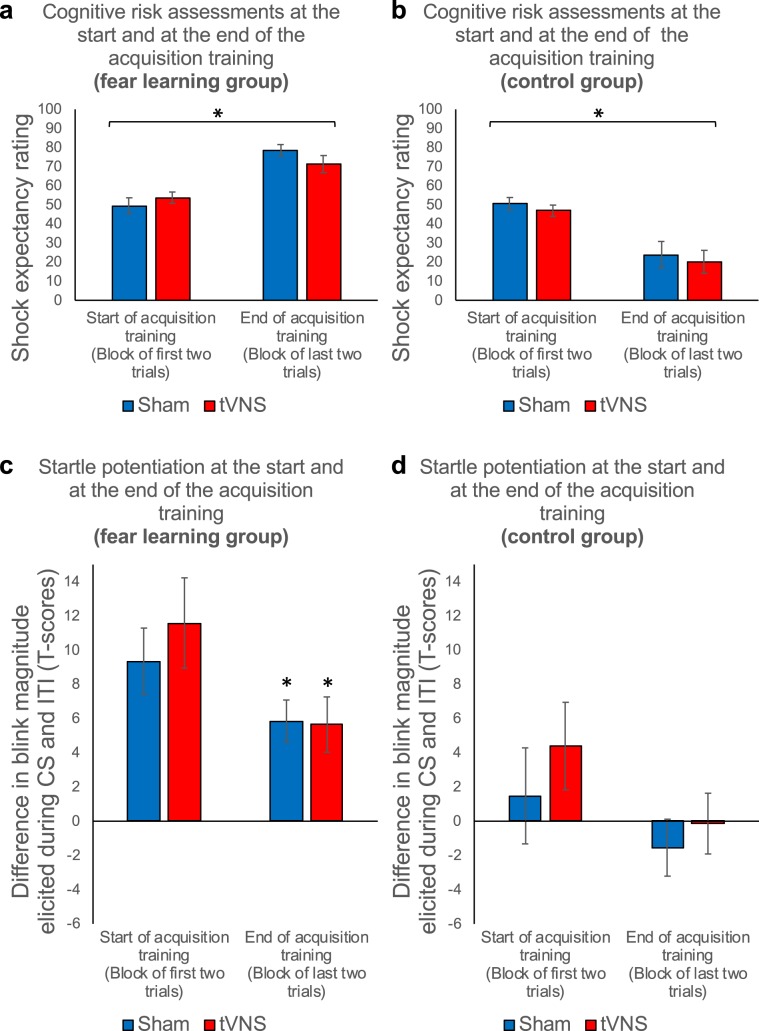
Paired presentations of CS and US result in stable conditioned fear responses (fear learning group) in comparison to explicitly unpaired presentations of CS and US (control group). Upper Panel (**a** and **b**): Mean shock expectancy ratings at the start of acquisition training (first two trials averaged) and at the end of the acquisition training (last two trials averaged) for the sham stimulation (*blue*) and tVNS condition (*red*) in the fear learning group (**a**: left) and in the control group (**b**: right). Error bars represent the standard error of the means (SEM). Participants of the fear learning group, receiving paired presentations of the CS and the electrical shock, showed an increase in rated shock expectancy from the start to the end of acquisition training. Participants of the control group, receiving explicitly unpaired presentations of the CS and the electrical shock, showed a decrease in rated shock expectancy from the start to the end of acquisition training. Lower Panel (**c** and **d**): Mean startle potentiation scores (standardized (T-scores) blink magnitudes elicited during the CS minus standardized (T-scores) blink magnitudes elicited during the inter-trial intervals) averaged across the magnitudes at the start of acquisition (first two probes in either condition (CS and ITI)) and at the end of acquisition training (last two probes in either condition (CS and ITI)). Potentiation scores are presented for sham stimulation (*blue*) and the tVNS condition (*red*) in the fear learning group (**c**: left) and in the control group (**d**: right). Again, error bars represent SEM. Participants of the fear learning group, receiving paired presentations of the CS and the electrical shock, showed stable fear potentiated startle responses at the end of the acquisition training. Participants of the control group, receiving explicitly unpaired presentations of the CS and the electrical shock, did not show fear potentiated startle responses at the end of the acquisition training.

Acquisition training established a reliable fear response in all response systems in the fear learning group but not in the control group. This was indicated by increasing shock expectancy ratings from the start to the end of acquisition training and increased startle potentiation during CS presentations relative to the ITI at the end of the acquisition training in the fear learning, but not in the control group, which in contrast showed decreasing shock expectancy ratings and a lack of potentiation of the startle response during the CS (trials × group, *F*_1,76_ = 71.13, *P* < 0.001, *η*^2^_*p*_ = 0.48; see Fig. 2a, b; potentiation × group, *F*_1, 228_ = 20.08, *P* < 0.001, *η*^2^_*p*_ = 0.08; see Fig. 2c, d). As expected, no differences were found between stimulation groups (tVNS vs. sham stimulation) in shock expectancy ratings (stimulation, stimulation × group, trials × stimulation, trials × stimulation × group, all *Fs* < 1, all *Ps* > 0.33) or fear potentiated startle responses (potentiation × stimulation, potentiation × trials × stimulation, potentiation × group × stimulation, potentiation × trials × group × stimulation, all *Fs* < 1.1, all *Ps* > 0.31).

### Transcutaneous vagus nerve stimulation facilitates initial extinction learning (session 2; extinction training)

**Figure 3:**
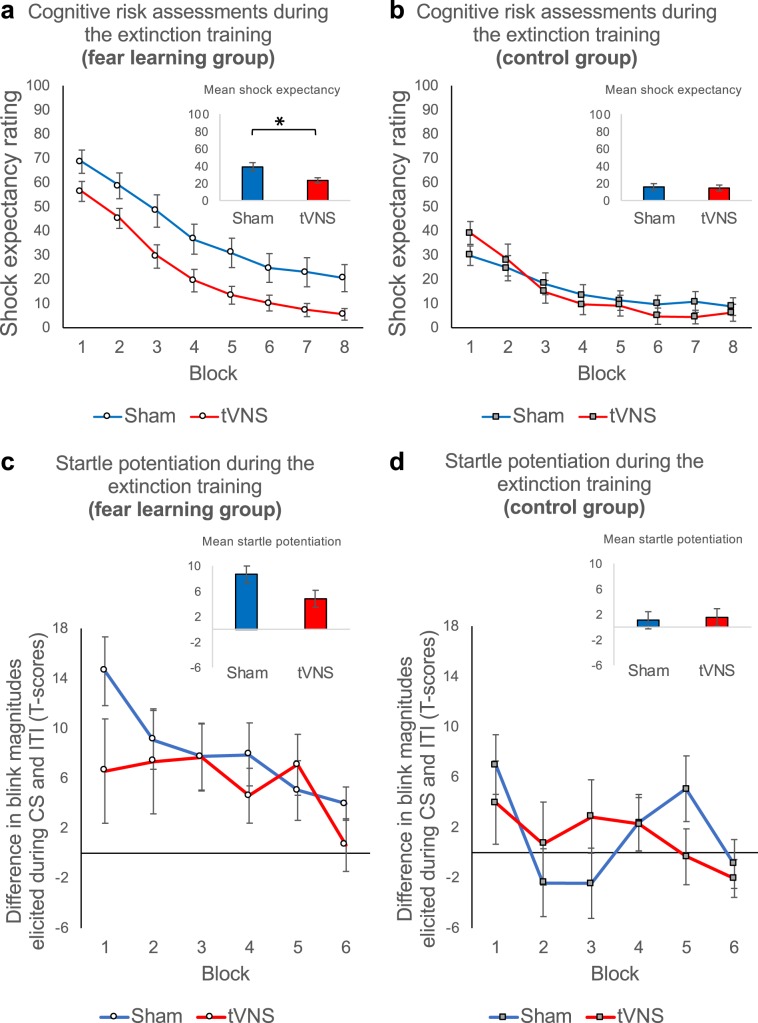
Transcutaneous vagus nerve stimulation facilitates fear extinction learning. Upper Panel (**a** and **b**): Mean shock expectancy ratings averaged across blocks of two extinction trials for the sham (*blue*) and tVNS condition (*red*) in the fear learning group (**a**: left) and in the control group (**b**: right). Error bars represent the standard error of the means (SEM). Participants of the fear learning group receiving tVNS showed facilitated reduction of fear in comparison to participants receiving sham stimulation (see **a**), indicated by a stronger decrease in shock expectancy ratings. Extinction processes in control group participants were not modulated by tVNS (see **b**). Lower Panel (**c** and **d**): Mean startle potentiation (standardized (T-scores) blink magnitudes elicited during the CS minus standardized (T-scores) blink magnitudes elicited during the inter-trial intervals) averaged across two probe stimuli presented during extinction training for the sham stimulation (*blue*) and tVNS condition (*red*) in the fear learning group (**c**: left) and the control group (**b**: right). Again, error bars represent SEM. We did not find effects of tVNS on the extinction of fear responses throughout the *whole* extinction training in neither group. However, we found that tVNS boosted extinction of fear responses in the fear learning group, but not the control group, late at the end of the extinction training (see Fig. 4).

Throughout the extinction training 24 hours later, shock expectancy ratings were still elevated in the fear learning relative to the control group (group, *F*_1,76_ = 16.90, *P* < 0.001, *η*^2^_*p*_ = 0.18; Fig. 3a, b). Moreover, blink magnitudes elicited during the CS were also significantly potentiated relative to the ITI in the fear learning, but not in the control group (potentiation × group, *F*_1,1058.90_ = 31.77, *P* < 0.001, *η*^2^_*p*_ = 0.03; see Fig. 3c, d). These results indicate a robustly consolidated fear memory.

Group differences diminished during course of extinction for expectancy ratings (group × trials, *F*_15,76_ = 7.86*, P* < 0.001, *η*^2^_*p*_ = 0.09) but remained stable for startle potentiation (potentiation × group × trials, *F* < 1), indicating that cognitive measures of fear adapt faster to changing stimulus outcome relationships compared to subcortically low-level indicators of defensive freezing.

As expected, tVNS modulated extinction learning in the fear learning but not in the control group. Shock expectancy ratings were significantly reduced in the fear learning group receiving tVNS compared to the sham condition (stimulation, *F*_1,38_ = 7.13*, P* = 0.011 *η*^2^_*p*_ = 0.16; see Fig. 3a), while tVNS had no overall effect in the control group (stimulation, *F* < 1; see Fig. 3b). This differential effect of vagus nerve stimulation tended to be stronger during the first half of extinction (group × stimulation, *F*_1,76_ = 3.73, *P* = 0.057, *η*^2^_*p*_ = 0.05; see Fig. 3a), being strongest at the beginning of extinction training (significant interaction group × stimulation during trials 1–2; *F*_1,76_ = 5.99, *P* = 0.017, *η*^2^_*p*_ = 0.07; Fig. 3a), indicating rapid anxiolytic effects of tVNS right from the start of extinction training.

At the end of extinction training, differences in shock expectancy between the fear learning and control group were no longer evident in tVNS participants, while we found a trend for higher shock expectancy ratings in sham subjects of the fear learning group compared to sham-controls (trials 15–16; group, *F*_1,38_ = 3.08, *P* = 0.087, *η*^2^_*p*_ = 0.08).

**Figure 4:**
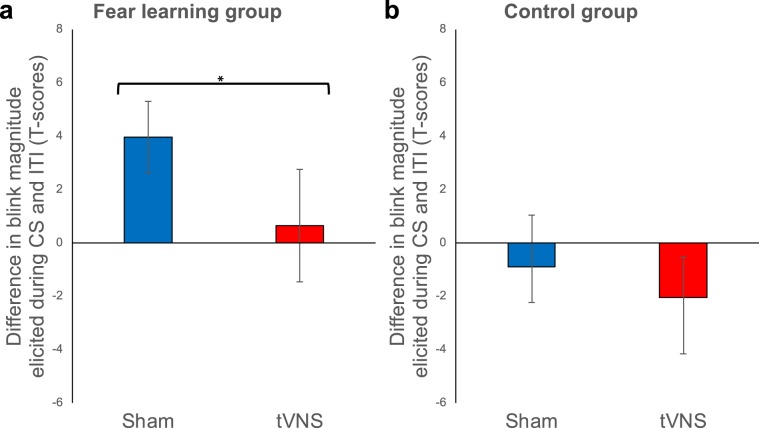
Transcutaneous vagus nerve stimulation boosts extinction of fear potentiated startle responses *late* during extinction training. Mean startle potentiation scores (standardized (T-scores) blink magnitudes elicited during the CS minus standardized (T-scores) blink magnitudes elicited during the inter-trial intervals) averaged across the magnitudes elicited by the last two probes in either condition (CS and ITI) during extinction training. Potentiation scores are presented for the sham stimulation (*blue*) and the tVNS condition (*red*) in the fear learning group (**a**: left) and the control group (**b**: right). Error bars represent SEM. At the end of the extinction training blink magnitudes were no longer potentiated during CS trials relative to ITI in the tVNS condition of the fear learning group (**a**: red bar). However, potentiation persisted for fear learning group receiving sham stimulation (**a**: blue bar). TVNS had no impact on fear potentiated startle responses in the control group (**b**: right) at the end of the extinction training.

Besides robust fear potentiated startle throughout extinction training (Fig. 3c, d), participants of the fear learning group receiving tVNS showed significantly reduced potentiation of the startle reflex *at the end* of extinction training in comparison to subjects receiving sham stimulation (see Fig. 4a, b). While blink magnitudes were no longer potentiated during CS trials relative to ITI for the last two extinction trials in the fear learning group receiving tVNS (trials 15–16; potentiation, *F* < 1; see Fig. 4a), potentiation persisted for fear learning group receiving sham stimulation (trials 15–16; potentiation, *F*_1,14.38_ = 11.88, *P* = 0.004, *η*^2^_*p*_ = 0.45, see Fig. 4a). As for cognitive risk assessments, differences in fear potentiated startle responses between the fear learning and control group were no longer evident in tVNS participants at the end of extinction training (last two probed trials; potentiation × group, *F*_1,35.10_ = 1.51, *P* = 0.227; Fig. 4a, b), while sham subjects of the fear learning group still showed higher fear potentiated startle responses compared to sham-controls (last two probed trials; potentiation × group, *F*_1,33.79_ = 5.91, *P* = 0.020, *η*^2^_*p*_ = 0.15; Fig. 4a, b). Thus, while declarative fear might have been reduced due to the anxiolytic effects of tVNS right from the start of extinction training, tVNS boosted the extinction of behavioral fear responses only late during extinction training.

### TVNS-paired extinction facilitates the subsequent extinction of conditioned fear responses after 24 hours (session 3; subsequent extinction test)

**Figure 5:**
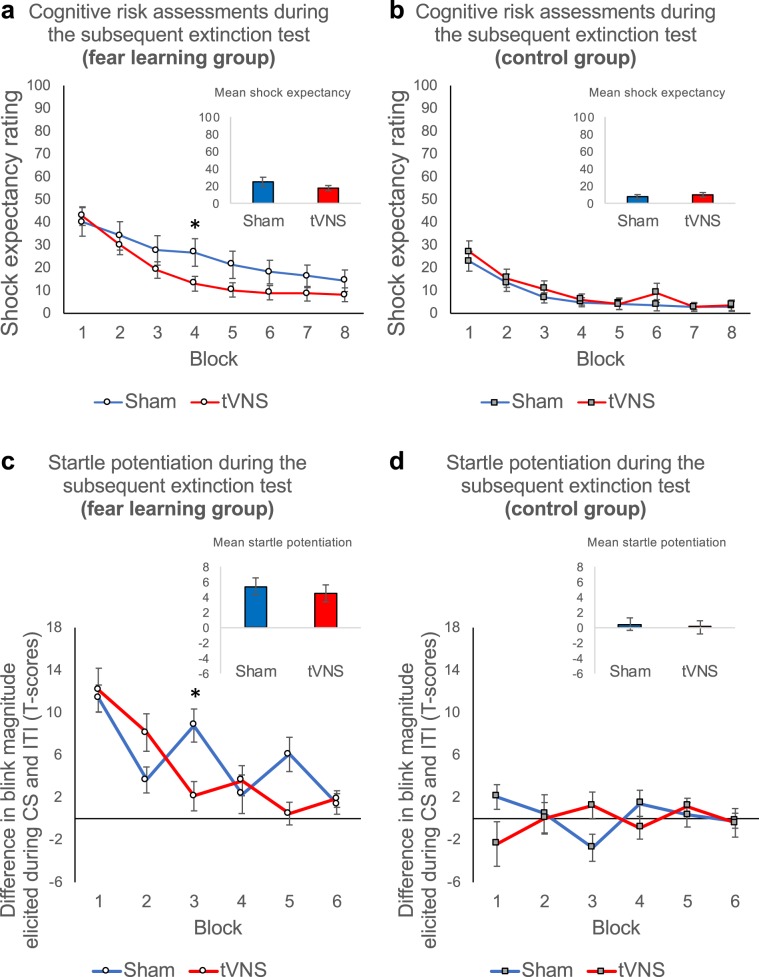
TVNS-paired extinction facilitates subsequent extinction of fear 24 hours later. Upper Panel (**a** and **b**): Mean shock expectancy ratings averaged across blocks of two subsequent extinction test trials for the sham stimulation (*blue*) and tVNS condition (*red*) in the fear learning group (**a**: left) and in the control group (**b**: right). Error bars represent SEM. Fear learning group participants who underwent extinction training under tVNS showed facilitated subsequent extinction 24 hours after the extinction training, indicated by a stronger decrease in shock expectancy ratings in the tVNS relative to the sham condition (see **a**). No such effect was observed in the control group (see **b**). Lower Panel (**c** and **d**): Mean startle potentiation (standardized (T-scores) blink magnitudes elicited during the CS minus standardized (T-scores) blink magnitudes elicited during the inter-trial intervals) averaged across two probe stimuli presented during the subsequent extinction test. Potentiation scores are presented for the sham stimulation (*blue*) and the tVNS condition (*red*) in the fear learning group (**c**: left) and the control group (d: right). Again, error bars represent SEM. Subsequent extinction of fear potentiated startle was accelerated by tVNS relative to sham (see **c**). No such effect was observed in the control group (see **d**).

Initial short-term recall of extinction memory was unaffected by tVNS, both for cognitive risk assessments (trials 1–2; stimulation, stimulation × group, all *Fs* < 1, all *Ps* > 0.49; see Fig. 5a, b) and potentiation of the startle responses (probes 1–2; potentiation × stimulation, potentiation × group × stimulation, all *Fs* < 1.20, all *Ps* > 0.27; see Fig. 5c, d). However, *during* the subsequent extinction test 24 hours later, we found that previous tVNS-paired extinction further facilitated extinction learning in the fear learning group, indicated by a stronger decrease in shock expectancy ratings in the tVNS relative to the sham condition (trials × stimulation, *F*_15,570_ = 2.34, *P* = 0.003, *η*^2^_*p*_ = 0.06; Fig. 5a). No such effect was observed in the control group (trials × stimulation, *F* < 1.; Fig. 5b). This effect was even more pronounced for potentiation of the startle response magnitudes, showing a significant group by stimulation by trials interaction (potentiation × trials × group × stimulation, *F*_11,1654.52_ = 2.02, *P* = 0.023, *η*^2^_*p*_ = 0.01; Fig. 5c, d). Thus, subsequent extinction of fear potentiated startle responses was facilitated by previous tVNS-paired extinction in the fear learning group relative to sham and this effect was not present in the control group (see also Supplementary Fig. 2). In sum, these findings indicate that previous tVNS-paired extinction facilitated subsequent extinction in both cognitive risk assessments and defensive brain reflex measures.

### TVNS-paired extinction results in the prevention of behavioral reinstatement of fear (session 3; reinstatement test I)

**Figure 6:**
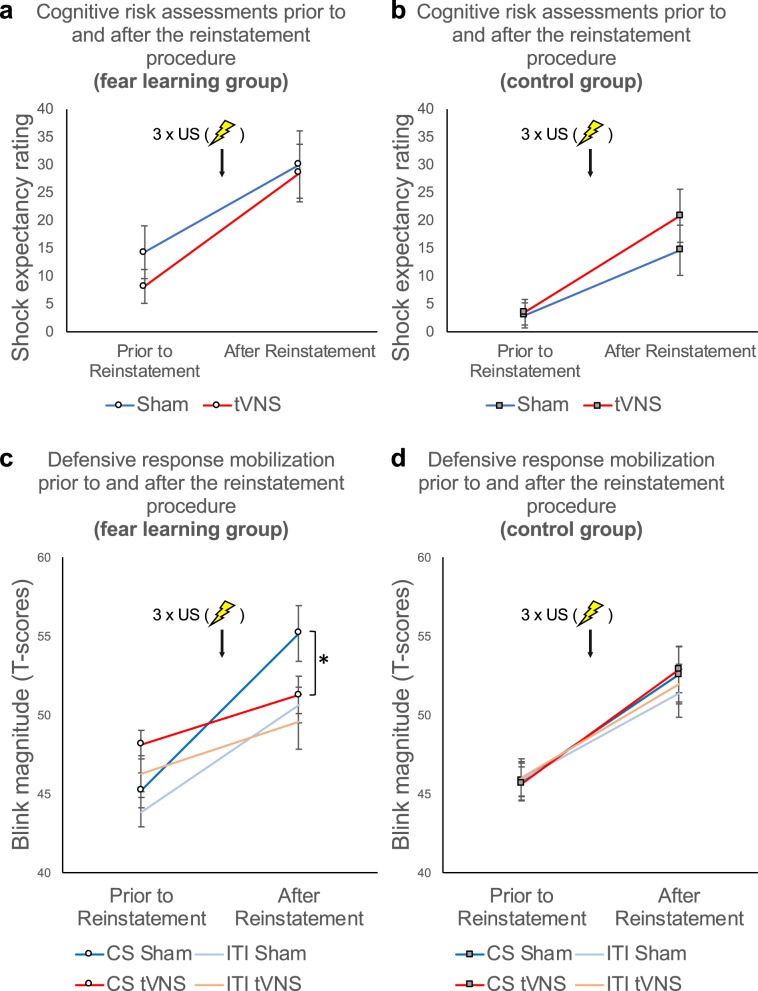
TVNS-paired extinction training results in the prevention of behavioral reinstatement of fear. Upper Panel (**a** and **b**): Mean shock expectancy ratings averaged across the last two subsequent extinction test trials prior to reinstatement and the first two trials following reinstatement for the sham stimulation (*blue*) and tVNS condition (*red*) in the fear learning group (a: left) and in the control group (b: right) Error bars represent SEM. TVNS-paired extinction did not affect the reinstatement of fear responses 24 hours later in neither group. Lower Panel (c and d): Blink magnitudes (standardized T-scores) elicited by the last two probes presented during the CS or the ITI prior to reinstatement for the sham stimulation (*blue shaded*) and the tVNS condition (*red shaded*) in the fear learning group (c: left) and the control group (d: right). Again, error bars represent SEM. TVNS-paired extinction resulted in a prevention of behavioral reinstatement of fear, as fear potentiated startle responses were only re-instated in the sham condition of the fear learning group, but not in the tVNS condition (see c). Reinstatement of fear was not modulated by tVNS in the control group (see d).

As depicted in Fig. 6, re-experience of the aversive event (three repetitive US presentations without CS) increased anxious apprehension and defensive response mobilization, as indicated by a significant increase in shock expectancy (Fig. 6a, b) and blink magnitudes (Fig. 6c, d) from the last two subsequent extinction test trials to the first two reinstatement test I trials (reinstatement, *Fs* = 48.72; 79.53; all *P*s < 0.001, all *η*^2^_*p*_ > 0.26 for shock expectancy ratings and blink magnitudes, respectively). Previous tVNS-paired extinction training did not affect the reinstatement effect for shock expectancy ratings (trials × stimulation, trials × stimulation × group, all *Fs* < 1.21, all *Ps* > 0.27). However, tVNS-paired extinction training resulted in significantly reduced startle response sensitization relative to the sham condition in the fear learning group but not in the control group (reinstatement × group × stimulation, *F*_1,224.56_ = 4.29, *P* = 0.039, *η*^2^_*p*_ = 0.02; Fig. 6c, d). Moreover, while potentiation of the blink magnitudes during CS presentations relative to ITIs was reinstated in the sham condition (potentiation, *F*_1,18_ = 5.75, *P* = 0.028, *η*^2^_*p*_ = 0.24; Fig. 6c), extinction of fear potentiated startle survived reinstatement in the tVNS group (potentiation, *F*_1,18.58_ = 1.11, *P* = 0.31; Fig. 4d).

After reinstatement, during the following reinstatement test I phase, shock expectancy ratings were still elevated in the fear learning group relative to the control group (group, *F*_1,76_ = 5.22, *P* = 0.025, *η*^2^_*p*_ = 0.06; Supplementary Fig. 1). Moreover, blink magnitudes were significantly potentiated in the fear learning but not in the control group (potentiation × group, *F*_1,1644.79_ = 9.59, *P* = 0.002, *η*^2^_*p*_ = 0.006; Supplementary Fig. 2), indicating that the fear memory trace was still evident.

### TVNS-paired extinction facilitates long-term recall of extinction memory (session 4; long-term extinction recall test and reinstatement test II)

**Figure 7:**
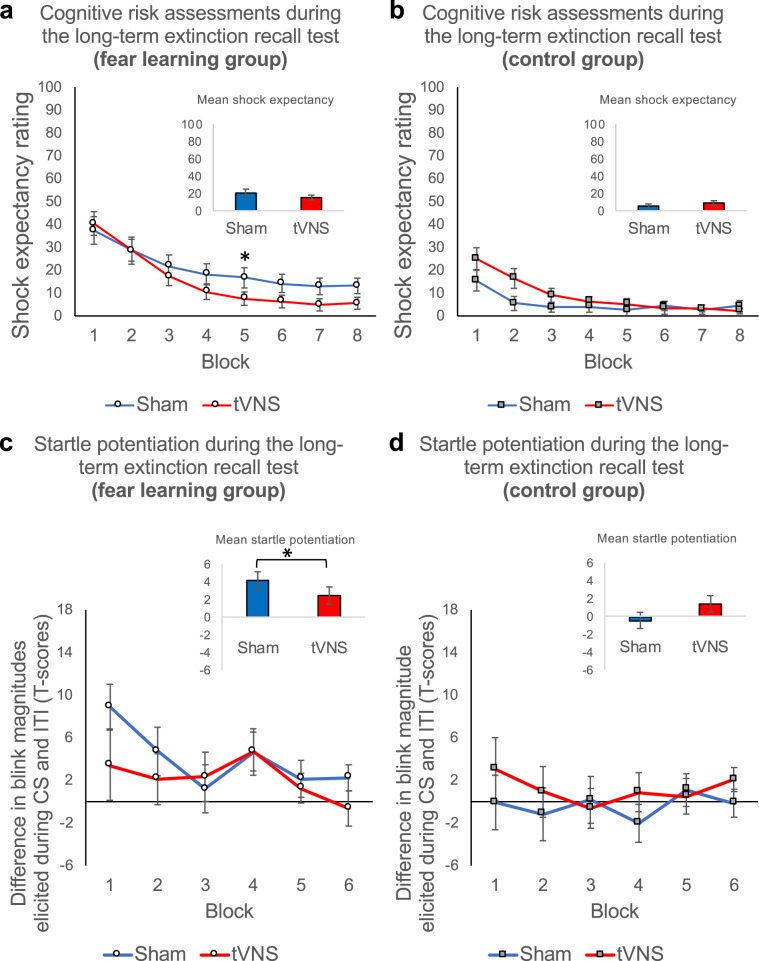
TVNS-paired extinction facilitates long-term recall of fear extinction memory (28 days after the subsequent extinction test). Upper Panel (**a** and **b**): Mean shock expectancy ratings averaged across blocks of two long-term extinction recall test trials for the sham stimulation (*blue*) and tVNS condition (*red*) in the fear learning group (**a**: left) and in the control group (**b**: right). Error bars represent SEM. Fear learning group participants who underwent extinction training under tVNS showed accelerated long-term extinction recall extinction 28 days after the subsequent extinction test, indicated by a stronger decrease in shock expectancy ratings in the tVNS relative to the sham condition (see **a**). No such effect was observed in the control group (see **b**). Lower Panel (**c** and **d**): Mean startle potentiation (standardized (T-scores) blink magnitudes elicited during the CS minus standardized (T-scores) blink magnitudes elicited during the inter-trial intervals) averaged across two probe stimuli presented during the long-term extinction recall test. Potentiation scores are presented for the sham stimulation (*blue*) and the tVNS condition (*red*) in the fear learning group (**c**: left) and the control group (**d**: right). Again, error bars represent SEM. Fear learning group participants who underwent extinction training under tVNS showed accelerated long-term extinction recall extinction 28 days after the subsequent extinction test, indicated by a stronger decrease in fear potentiated startle responses in the tVNS relative to the sham condition (see **a**). No such effect was observed in the control group (see **b**).

Even after 28 days, the fear memory trace was still evident, indicated by elevated shock expectancy ratings and startle potentiation in the fear learning group relative to the control group (group and potentiation × group, *Fs* = 11.02; 11.05, all *P*s < 0.05, all *η*^2^_*p*_ > 0.007 for shock expectancy ratings and blink magnitudes, respectively; see Fig. 7a–d). Initial long-term recall of extinction memory was unaffected by tVNS, both for cognitive risk assessments (trials 1–2; stimulation, stimulation × group, all *Fs* < 1.79, all *Ps* > 0.18; see Fig. 7a) and potentiation of the startle responses (probes 1–2; potentiation × stimulation, potentiation × group × stimulation, all *Fs* < 2.45, all *Ps* > 0.12; see Fig. 7c). Importantly, participants who received tVNS, but not sham stimulation on day 2, showed better recall of fear extinction memory *during* the long-term extinction recall test, 28 days after session 3. This was indicated by stronger reduction of shock expectancy in the fear learning group receiving tVNS compared to the sham condition (trials × stimulation, *F*_15,570_ = 1.97, *P* = 0.016, *η*^2^_*p*_ = 0.05; Fig. 7a). Moreover, extinction of the potentiation of the startle response magnitudes in the fear learning group was again facilitated by previous (4 weeks ago) tVNS relative to the sham condition (potentiation × group × stimulation, *F*_1,1668.68_ = 5.09, *P* = 0.024, *η*^2^_*p*_ = 0.003; Fig. 7c, d).

After 28 days, re-experience of the aversive event also led to a significant defensive sensitization, indicated by a significant increase in shock expectancy and blink magnitudes from the last two long-term extinction recall test trials to the first two reinstatement test II trials in the fear learning and the control group (reinstatement, *Fs* = 25.33; 13.27; all *P*s < 0.001, all *η*^2^_*p*_ > 0.05 for shock expectancy ratings and blink magnitudes, respectively; Supplementary Figs. 1 and 2). Importantly, differences between the fear learning group and the control group were no longer significant in shock expectancy ratings (group, *F*_1,76_ = 3.64, *P* = 0.060, *η*^2^_*p*_ = 0.01; Supplementary Fig. 1) and startle potentiation (potentiation × group, *F* < 1; Supplementary Fig. 2), indicating fully extinguished fear in the fear learning group.

## Discussion

Using a multiple-day single-cue conditioning and extinction paradigm, adapted to the experimental protocols used in animal studies^18,19^, we found that an extinction training paired with transcutaneous vagus nerve stimulation resulted in both rapid anxiolytic effects as well as a facilitation of fear extinction learning in comparison to a sham stimulation. During the extinction training, anxiolytic effects were observed for cognitive indicators of anxious apprehension (shock expectancy ratings), while tVNS facilitated the extinction of subcortically mediated indicators of defensive freezing (fear potentiated startle response). Moreover, tVNS boosted the subsequent extinction, as well as long-term recall of extinction memory and also prevented reinstatement of fear. Thus, our findings provide evidence, that tVNS not only facilitates the inhibition of fear itself, but is also capable of facilitating further subsequent extinction of cognitive and behavioral indices of conditioned fear and long-term recall of extinction memory in humans.

It has been repeatedly proposed that transcutaneous vagus nerve stimulation results in both anxiolytic effects and enhanced cognitive flexibility in humans and rodents^39–42^. In fact, our results support the hypothesis, that tVNS promotes both an overall reduction of cognitive risk assessments and a flexible cognitive adaptation to changing aspects of the environment. Accordingly, active tVNS significantly reduced cognitive risk assessments relative to sham stimulation, when a cue was no longer predicting an aversive event. As this effect was strongest early during extinction learning and was even evident right from the start of extinction training, we may assume rapid anxiolytic effects, as proposed by previous rodent research^41^. However, even more remarkable, although the initial spontaneous recovery of fear after a passage of 24 hours has not been prevented by a tVNS-paired extinction training, it in fact promoted flexible cognitive adaptation and further facilitated the extinction of cognitive risk assessments 24 hours later during a subsequent extinction test.

Extending these findings, that are also in line with previous human data from Burger and coworkers^23,24^, we also observed long-term effects of tVNS on recall of extinction memory. Again, although a spontaneous recovery of fear after 28 days was not prevented by tVNS-paired extinction, these new finding suggest that tVNS may also facilitate the reduction of long-term risk assessments by consolidating the extinction memory that a cue is no longer associated with an aversive event. Since elevated expectations of aversive events in a certain context seem to be important for motivating avoidance behavior^43,44^ transcutaneous vagus nerve stimulation might be an important adjunct to cognitive-behavioral exposure-based therapy, aimed to reduce persistent avoidance behavior in patients with anxiety disorders.

In contrast to previous human research^23–25^, we found that tVNS also promoted extinction of fear potentiated startle responses, thus in line with animal findings showing that stimulation of vagal afferents promoted extinction of defensive freezing^18–20^. One reason for this first successful translation to human research might be, that we used individually adjusted intensity of vagal stimulation, presumably coping better with participants’ individual differences in skin resistance. More importantly, we used a simple learning task highly comparable to the one used in rodent research^18–20^. In fact, using a single-cue conditioning and extinction paradigm, we found that tVNS facilitated inhibition of fear potentiated startle during extinction training and, more remarkably, during a subsequent extinction test after 24 hours. Most strikingly, tVNS improved recall of extinction memory when tested after 28 days. While the fear learning group still showed elevated shock expectancy ratings and potentiated startle during the conditioned cue in the sham condition, no such effect was observed for the fear learning group receiving tVNS during initial extinction training. Finally, behavioral reinstatement of fear was not only inhibited, but even prevented by tVNS, indicating enhanced consolidation of extinction memory due to vagal stimulation.

Importantly the extinction of conditioned startle reflex potentiation took longer compared to the reduction of shock expectancy ratings, suggesting that the extinction process of subcortically mediated defensive behaviors might take more time than learning on a cognitive level, that the environment has changed. This is particularly important for a better understanding of the mechanisms, that might be responsible for the behavioral return of fear. The current data suggest, that it takes more trials to extinguish defensive action dispositions than cognitive risk assessments. Correspondingly, behavioral fear reducing effects of tVNS took longer to evolve during extinction training, indicating that behavioral fear reducing effects of tVNS boost extinction learning late during extinction training. These data therefore suggest, that while tVNS might have an inherent anxiolytic effect on cognitive fear responses, this effect may not be evident for low level behavioral indices of fear responses. It is therefore even more remarkable, that the current findings indicate that tVNS facilitates long-term extinction of defensive behavior in humans.

We want to further emphasize, that tVNS did not affect the startle responses of the control group during the ITIs during extinction training and subsequent test sessions. Since the control group received US presentations that were explicitly unpaired with the CS, such procedure might also have resulted in increased contextual anxiety in this group, as the US occurred unpredictably during the ITI^45^. However, as tVNS resulted in stronger effects in the fear learning group, we may assume that the extinction enhancing effects of tVNS might be specific to the extinction of cued fear, and may not affect the extinction of contextual anxiety, thus supporting previous work by Genheimer and coworkers^46^.

Elaborating the mechanisms of action, animal data suggest that the stimulation of vagal afferents results in specific noradrenergic activation of the basolateral amygdala (BLA) and the ventromedial prefrontal cortex (vmPFC)^15–17^. Human research indicates that tVNS also activates the BLA, the vmPFC and also the locus coeruleus^21,47^, the central hub for releasing noradrenaline located in the brainstem^48,49^. These circuits are also involved in extinction of conditioned fear responses^1,9,12,50^. Thus, we may assume that tVNS facilitates fear extinction by augmentation of noradrenergic signaling in the BLA and the vmPFC, presumably resulting in subsequent inhibition of the central nucleus of the amygdala by their projections to GABAergic intercalated cells^4–6^. In fact, animal research has shown that synaptic plasticity in the vmPFC-BLA pathway – probably critical for long-term fear inhibition - is indeed modulated by vagus nerve stimulation^18–20^. As our data also show improved (long-term) extinction of conditioned fear after tVNS, the same mechanism of action might work in humans.

The current data suggest that tVNS may be a promising adjuvant for exposure-based treatments, the preferred strategy in treating anxiety disorders. Patients with anxiety disorders do show impairments in extinction learning^51,52^, probably contributing to the high percentage of relapses after exposure-based treatments and therapy non-responders^33,34,53,54^. As tVNS resulted in anxiolytic effects as well as a facilitation of extinction learning and long-term extinction recall, transcutaneous vagus nerve stimulation may be helpful to reduce the proportion of therapy non-responders but might also be helpful to reduce relapses after successful therapy.

## Methods

### Subject details and ethical approval

Eighty subjects (23 men, 57 women), mainly chosen from the student population of the University of Greifswald, ranging in age from 18 to 34 (*M* = 22.75, SD = 3.46), participated in the study.

Each participant gave her/his informed consent. All participants either received partial course credits or monetary reward (34 €). This study was approved by the ethical committee of the German Society for Psychology (“Deutsche Gesellschaft für Psychologie; DGPs”). The experiment was performed in accordance to relevant guidelines and regulations.

### Eligibility Criteria

Each participant underwent a screening interview by phone to check for in- and exclusion criteria. All participants were assessed to be 18–35 years old with a body-mass-index in normal range (18.5 kg/m^2^ to 27 kg/m^2^) and to be free from any current or previous medical condition or mental disorder (including nicotine addiction) that would have affected any of the outcome measures or would have contraindicated the application of tVNS (i.e., pregnancy checked by a pregnancy test prior to the study or electrical implants).

### Randomization

Eligible participants were then randomly assigned to one of four groups: a fear learning group (*n* = 40) receiving either tVNS (*n* = 20) or sham stimulation to the earlobe (*n* = 20) and a control group (*n = *40) receiving either tVNS (*n* = 20) or sham stimulation to the earlobe (*n* = 20). The sample size was determined based on previously published articles in the field of fear conditioning and extinction^55^.

### Single-cue fear conditioning paradigm

It was recently noted, that fear conditioning and extinction protocols often substantially differ between rodent and human research, which in consequence hampered the scientific transfer^56^. We therefore used a multiple-day single-cue fear conditioning and extinction paradigm as it is commonly used in rodent research (for further details, see **Experimental procedure**, see also Fig. 1).

## Stimulus Materials

### Conditioned stimulus (CS)

A blue pentagon on a black background, displayed for 7.135 s on a 24-inch computer monitor with a pixel resolution of 1024 × 768, served as conditioned stimulus (see Fig. 1).

### Inter-trial interval (ITI)

A black screen, displayed for either 12, 14 or 16 s (*M* = 14 s) on a 24-inch computer monitor with a pixel resolution of 1024 × 768, served as inter-trial interval (see Fig. 1).

### Unconditioned stimulus (US; electrical stimulation)

An individually adjusted electrical shock served as unconditioned stimulus and was delivered by an S-48K stimulator (Grass instruments) and characterized by a 625 ms period of stimulation, consisting of 125 single pulses with a duration of 2 ms each and a 3 ms break between the stimuli. The average US intensity for the tVNS condition was 3.41 mA (SD = 1.53) and 3.44 mA (SD = 1.47) for the sham condition. No significant difference could be observed in US intensities between the tVNS and sham condition (stimulation, group × stimulation, all *Fs* < 1.09, *P* > 0.30).

### Acoustic startle probe

A binaurally presented burst of white noise (95 dB, duration of 50 ms, rise/fall time < 1 ms) served as the acoustic startle probe stimulus.

### Transcutaneous vagus nerve stimulation (tVNS)

The tVNS stimulation device (CMO2, Cerbomed, Erlangen, Germany) was located in the left auricle where two titan electrodes were placed in one of two positions: In the group receiving active vagus nerve stimulation (tVNS) the electrodes were placed in the cymba conchae, an area exclusively innervated by the auricular branch of the vagus nerve (ABVN)^22^, hence stimulating only vagal afferents. In the group receiving a sham stimulation the electrodes were positioned in the center of the ear lobe, an area free of vagal innervation since it is innervated by the great auricular nerve (GAN). The stimulation was delivered during the whole extinction training (session 2), applying a 30 s ON and 30 s OFF procedure with a pulse width of 200–300 μs at a rate of 25 Hz. To ensure the activation of the ABVN or GAN, the stimulus intensity of the stimulation was set to be clearly perceived but with no associated discomfort. All participants therefore underwent a stimulation workup prior to the first extinction training on session 2, where they adjusted the stimulation intensity on their own to be perceptible but below the pain threshold. The average stimulation intensity for both groups was 2.28 mA (SD = 1.13) for the tVNS and 2.53 mA (SD = 1.11) for the sham condition. No significant difference could be observed in stimulation intensities between the tVNS and sham condition (stimulation, group × stimulation, all *Fs* < 1.31, all *Ps* > 0.25). Stimulation was administered for approximately 10 min.

## Experimental Procedure

### Experimental setting

During each session the participants sat in a dimly lit, sound-attenuated room 1.45 m in front of a computer monitor. First sensors were attached for recording physiological signals as well as the electrodes to deliver the electrical shock (at the non-dominant hand’s wrist). A shock workup procedure followed, in which the intensity of the US was individually set at a level the participant defined to be clearly unpleasant, but not painful^57^. Then participants were instructed that during the subsequent learning sessions (acquisition training, extinction training, subsequent extinction test with following reinstatement test I or long-term extinction recall test with following reinstatement test II), the CS, the US and acoustic startle probes may be presented at any time without any explicit information with regard to the contingencies. Each learning session began with a 74 s startle habituation phase, where 6 acoustic startle probes (95 dB, duration 50 ms) were presented repetitively to adapt startle magnitudes to a stable baseline.

### Acquisition training (session 1)

During the acquisition training the CS was presented 16 times, with a respective duration of 7.135 s and an inter-trial interval of 12, 14 or 16 s (*M* = 14 s, see stimulus materials). To establish a robust and reliable fear response and to increase resistance to extinction, the electrical shock (US) was delivered during the CS in 12 out of 16 trials in the fear learning group (reinforcement rate: 75%). Electrical shocks lasted for 625 ms and were delivered 6.5 s after CS-onset. In the control group 12 shocks were explicitly unpaired with the CS presentation and delivered during the inter-trial intervals (ITI) to ensure that sensitization effects did not differ between groups (US onset of 3, 4, 5, 6, 7, 8, 11 or 12 s after ITI onset (*M* = 6.98 s)). Acoustic startle probes were presented during the CS in 12 out of 16 trials in both the fear learning and control group either 4.5, 5 or 5.5 s after CS onset (*M* = 5 s). Moreover, 12 probes were presented during the inter-trial intervals in 12 out of 16 trials in both the fear learning and control group either 6, 7 or 8 s after ITI onset (*M* = 7 s).

### Extinction training (session 2)

The second day of investigation took place 24 +/− 4 hours after the acquisition training. After refitting the electrodes for physiological recording and US-delivery, tVNS/sham stimulation was applied. The participants were instructed to adjust the transcutaneous vagus nerve/sham stimulation intensity to be perceptible without being painful starting with an intensity of 0.1 mA. Participants were required to rate their subjective sensation of the stimulation intensity after each adjustment of 0.1 mA on a visual 11-point scale, ranging from nothing (0), light tingling (3), strong tingling (6) to painful (10). The tVNS workup lasted until the participants reported a “tingling” sensation of 8, after which they underwent a 30 s ON and 30 s OFF stimulation protocol to experience the stimulation as it would be during the extinction training^47^. If the participant still reported a sensation of 8 after the protocol, the adjusted intensity was used during extinction training. Then participants were instructed that the session would begin with a 3-minute period to adjust to the tVNS and sham stimulation before CSs, USs and acoustic startle probes might be presented again as during the previous day. Again, no explicit references to the contingencies were made, i.e., participants were not informed that the US was no longer going to be delivered during the following extinction training. The CS was presented 16 times without any US. Acoustic startle probes were presented similar to session 1 (see Fig. 1).

### Subsequent extinction test and reinstatement test I (session 3)

The subsequent extinction test took place 24 +/- 4 hours after the extinction training. After electrodes for physiological recordings and stimulation were attached, participants were again instructed as prior to the extinction training. During the subsequent extinction test the CS was presented 16 times without any presentation of the US. Then the reinstatement procedure (reinstatement test I) followed during which the US was presented repetitively three times without any CS. However, the background color of the monitor changed to grey in order to exclude conditioning to the background color of the monitor during the ITI. Afterwards the CS was presented again for 16 trials without any presentation of the US (see Fig. 1).

### Tests for long-term extinction recall and reinstatement test II (session 4)

Approximately 28 days (*M* = 28.26, SD = 3.46) after the third session, with a minimum interval of 21 days, the same procedure as on session 3 was repeated (see Fig. 1) to assess long-term recall of extinction memory.

## Assessments

### Shock expectancy ratings

Prior to each CS presentation, participants were asked to rate their subjective expectancy of US occurrence during the upcoming CS presentation on a continuous 11-point rating scale (ranging from “0%” to “100%”) by shifting a red cursor and pushing the left mouse button (see Fig. 1). During this rating procedure, the CS was presented in smaller size and participants were instructed to rate the probability, that this cue would be followed by the shock during the upcoming trial. This procedure is very much comparable to the clinical procedure during exposure therapy, during which the patient is first asked to rate the probability that the central concern might become true (e.g., fainting) before the exposure exercise begins. Three seconds after completing the rating the conditioned stimulus is presented in full size on the screen. Thus, we made sure that the physiological fear response is not affected by a parallel cognitive evaluation task.

### Startle eyeblink response

To measure the eyeblink component of the startle response as an amygdala-dependent indicator of defensive freezing, the electromyographic activity was recorded using two electrolyte filled (Marquette Hellige, Freiburg, Germany) Ag/AgCl miniature surface electrodes (3 mm diameter, Sensormedic, Yorba Linda, CA) attached over the orbicularis oculi muscle underneath the left eye. The EMG signal was amplified by a Coulbourn S75–01 amplifier and filtered with a 30 Hz high-pass and a Kemo LEM-VBF8–03 400 Hz low-pass filter (smoothing the rectified signal with a time constant of 10 ms). Digital sampling was carried out at a rate of 1000 Hz between 100 ms before and 400 ms after the onset of the startle probe. Startle eyeblink responses were scored semi-automatically using a computer program^58^, identifying blink onset and peak amplitude. Valid startle response was scored, if blinks started 20–120 ms after the onset of the acoustic startle probe and peaked within 150 ms with a minimum amplitude of 1.954 μV. If no blink was detected in the defined time window, the trials were scored as zero responses. Trials with clear movement artifacts or excessive baseline shifts were set as missings^59^. Raw blink magnitudes were standardized individually for each participant using a z-score transformation. Subsequently, these standardized responses were converted to T-scores (50 + (z × 10)) for each participant individually. Thus, we made sure that every participant contributed equally to the groups’ mean. As the stimulation device for tVNS and sham stimulation produced some noise during extinction training on session 2, only trials were scored, if no stimulation artifacts could be obtained. For acquisition training (session 1), 2.2% of all probed trials were set as missing (*M* = 0.66) and 0.5% were scored as zero responses (*M* = 0.15). For extinction training (session 2), on average 31.2% of all probed trials were set as missing (*M* = 9.38; higher rate of missings due to tVNS induced noise) and 0.3% were scored as zero responses (*M* = 0.09). For the subsequent extinction test (session 3), 4.1% of all probed trials were set as missing (*M* = 1.25) and 1.4% were scored as zero responses (*M* = 0.43). For short-term reinstatement test I (session 3), 4.5% of all probed trials were set as missing (*M* = 1.35) and 3.2% were scored as zero responses (*M* = 0.96). For long-term extinction recall test (session 4), 3.5% of all probed trials were set as missing (*M* = 1.06) and 1.4% were scored as zero responses (*M* = 0.43). For reinstatement test II (session 4), 4.4% of all probed trials were set as missing (*M* = 1.33) and 3.5% were scored as zero responses (*M* = 1.05). So, except for extinction training (during which stimulation induced more noise) proportion of missing data were below 5% during all phases of the experiment.

### Electrocardiogram and skin conductance level

We also recorded an electrocardiogram (ECG) and the skin conductance level (SCL) for all experimental sessions. However, the focus of this manuscript firstly lies on the translation of the extinction enhancing effects of vagal stimulation on behavioral indicators of fear, which were found in rodents^18–20^. Second, we wanted to replicate previous results in humans, showing extinction enhancing effects of tVNS on cognitive indicators of fear^23–25^. Thus, the ECG and SCL data are out of this manuscript’s scope.

### Statistical analyses and graphical representation

Shock expectancy ratings and startle response magnitudes were analyzed using linear mixed regression models with only fixed effects included and an underlying compound symmetry covariance matrix, offering a flexible and powerful analysis of the repeated measures data with missing values. This type of linear mixed regression was chosen, because it is identical to a repeated-measures ANOVA model, the common strategy of analysis for physiological data with multiple time points, with the advantage of also including participants with missing values^60,61^, thus, providing stronger comparability to previous fear conditioning studies. Group (fear learning vs. control) and Type of Stimulation (tVNS vs. sham) served as between-subject factors with Trials as within-subject factor. For analyses of startle response magnitudes, the factor Potentiation (CS vs. inter-trial intervals) was included as an additional within-subject factor. Partial Eta-squared was computed following recommendations by Lakens^62^. We conducted the statistical analyses using IBM SPSS Statistics 25. Microsoft Excel was used for creating the figures.

## Data and software availability

The data, that support the findings of this study, are available from the corresponding author (Christoph Szeska (christoph.szeska@uni-greifswald.de)) upon reasonable request.

## Supplementary information

Supplemental Information.
