# Supplementary material for: Promoting long-term inhibition of human fear responses by non-invasive transcutaneous vagus nerve stimulation during extinction training
Source: Sci Rep. 2020 Jan 30;10:1529. doi: 10.1038/s41598-020-58412-w (PMC6992620; doi:10.1038/s41598-020-58412-w)
Supplement: Supplementary file 1 — Supplemental Information. [file 41598_2020_58412_MOESM1_ESM.pdf]

Supplemental information

Promoting long-term inhibition of human fear responses by non-invasive transcutaneous vagus nerve stimulation during extinction training

Christoph Szeska*[1], Jan Richter[1], Julia Wendt[2], Mathias Weymar[2], & Alfons O. Hamm[1]

*Corresponding author

[1] University of Greifswald, Department of Physiological and Clinical Psychology / Psychotherapy, Franz-Mehring-Strasse 47, 17487 Greifswald, Germany

[2] University of Potsdam, Department of Biological Psychology and Affective Science, Karl-Liebknecht Strasse 24/25, 14476 Potsdam, Germany

Corresponding author:
Christoph Szeska
University of Greifswald
Franz-Mehring-Strasse 47
17487 Greifswald
Phone: +49 3834 420 3797
Fax: +49 3834 420 3763
Email: christoph.szeska@uni-greifswald.de

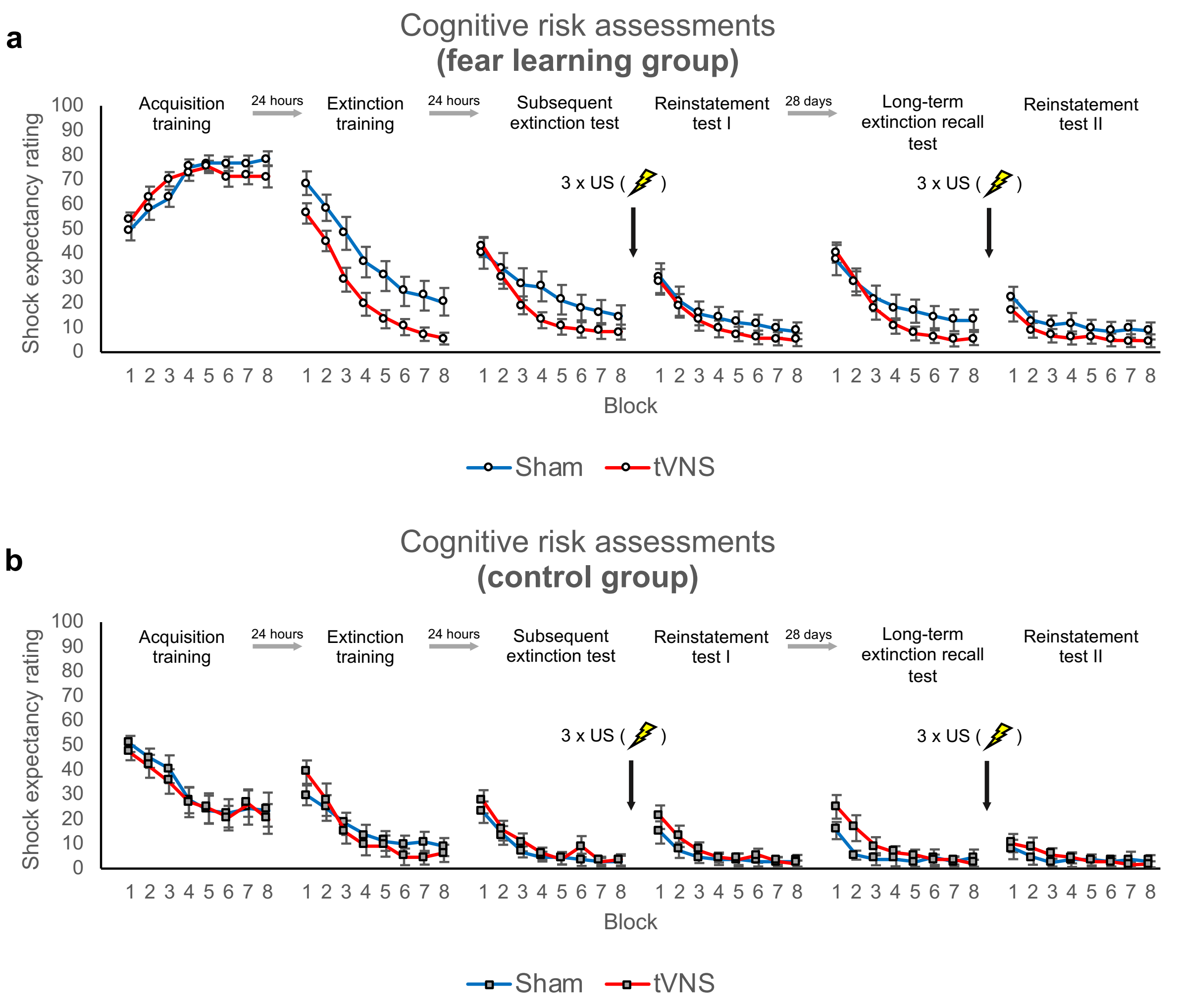

Supplementary Figure 1. Cognitive risk assessments during the multiple-day single-cue fear conditioning and extinction paradigm.

Mean shock expectancy ratings during CS trials averaged across blocks of 2 trials, during acquisition training (session 1), extinction training 24 hours later (session 2), the subsequent extinction test 24 hours after extinction training (session 3), reinstatement test I immediately following the subsequent extinction test (session 3), the long-term extinction-recall test 28 days after the subsequent extinction test (session 4) and reinstatement test II immediately following the long-term extinction recall test for the sham stimulation (*blue*) and tVNS condition (*red*) of the fear learning group (upper panel: a) and the control group (lower panel: b). Error bars represent SEM.

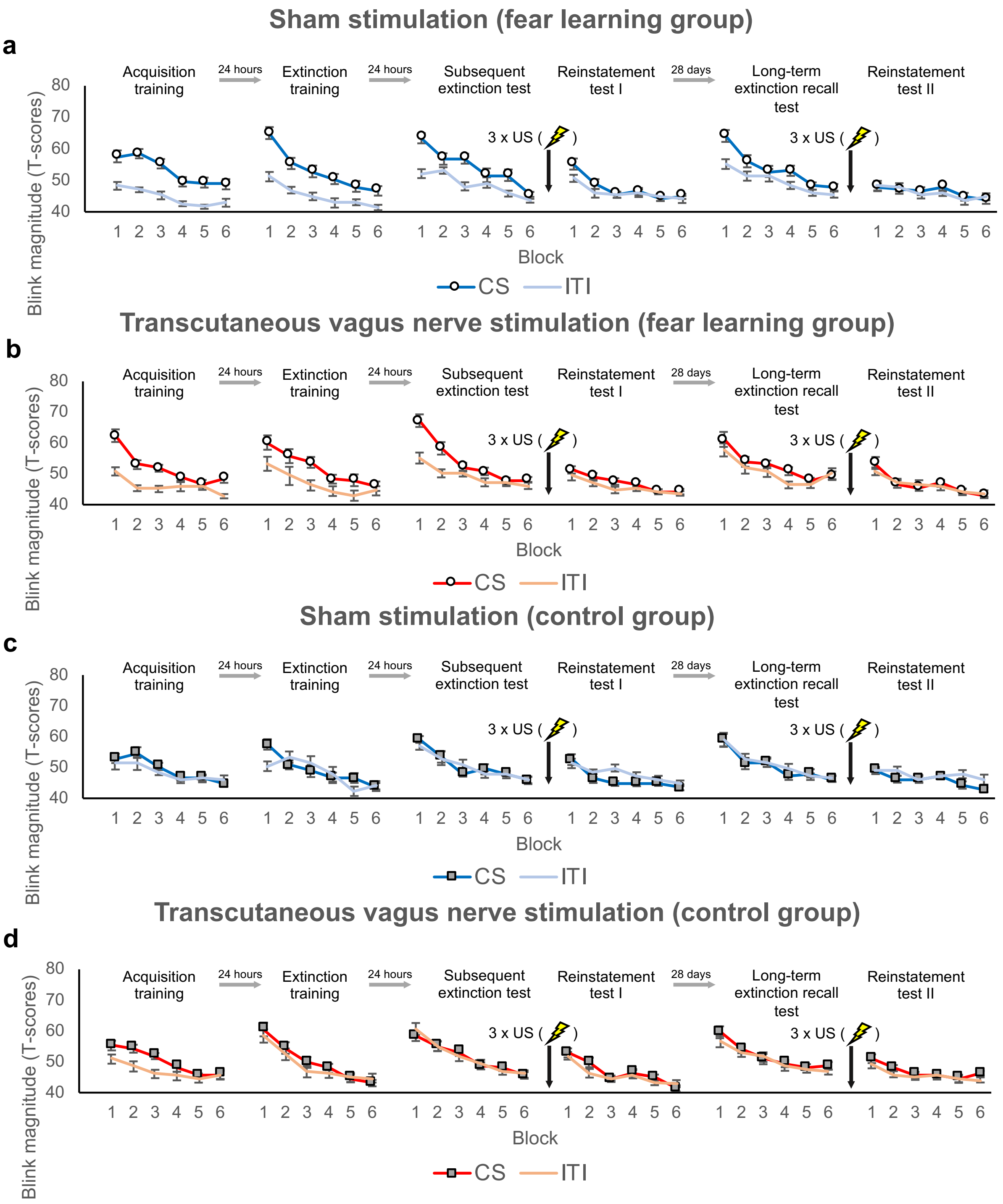
Sham stimulation (fear learning group)
a
Transcutaneous vagus nerve stimulation (fear learning group)
b
Sham stimulation (control group)
c
Transcutaneous vagus nerve stimulation (control group)
d
Acquisition training
24 hours
Extinction training
24 hours
Subsequent extinction test
Reinstatement test I
28 days
Long-term extinction recall test
Reinstatement test II
3 x US
Blink magnitude (T-scores)
Block
CS
ITI

Supplementary Figure 2. Startle responses during the multiple-day single-cue fear conditioning and extinction paradigm.

Mean blink magnitudes (T-scores) elicited during the CS and during inter-trial intervals averaged in blocks across two startle probes during acquisition training (session 1), extinction training 24 hours later (session 2), the subsequent extinction test 24 hours after extinction training (session 3), reinstatement test I immediately following the subsequent extinction test (session 3), the long-term extinction-recall test 28 days after the subsequent extinction test (session 4) and reinstatement test II immediately following the long-term extinction recall test for the sham stimulation (*blue shaded lines;* panels: a and c) and the tVNS condition (*red shaded lines;* panels: b and d). Error bars represent SEM.
